# Photocatalytic Degradation of Bacteriophages Evidenced by Atomic Force Microscopy

**DOI:** 10.1371/journal.pone.0053601

**Published:** 2013-01-03

**Authors:** Emrecan Soylemez, Maarten P. de Boer, Udom Sae-Ueng, Alex Evilevitch, Tom A. Stewart, May Nyman

**Affiliations:** 1 Mechanical Engineering Department, Carnegie Mellon University, Pittsburgh, Pennsylvania, United States of America; 2 Department of Physics, Carnegie Mellon University, Pittsburgh, Pennsylvania, United States of America; 3 Geochemistry, Sandia National Laboratories, Albuquerque, New Mexico, United States of America; Harbin Institute of Technology, China

## Abstract

Methods to supply fresh water are becoming increasingly critical as the world population continues to grow. Small-diameter hazardous microbes such as viruses (20–100 nm diameter) can be filtered by size exclusion, but in this approach the filters are fouled. Thus, in our research, we are investigating an approach in which filters will be reusable. When exposed to ultraviolet (UV) illumination, titanate materials photocatalytically evolve ^•^OH and O2^•−^ radicals, which attack biological materials. In the proposed approach, titanate nanosheets are deposited on a substrate. Viruses adsorb on these nanosheets and degrade when exposed to UV light. Using atomic force microscopy (AFM), we image adsorbed viruses and demonstrate that they are removed by UV illumination in the presence of the nanosheets, but not in their absence.

## Introduction

More than a billion people lack access to potable water and sanitation in the world [1]. Nanotechnology developments can address this critical problem by enhancing filtration techniques, which are effective and common but are susceptible to fouling. Nanocatalysts are effective in reducing fouling; they catalyze chemical reactions to remove bacteria, viruses and organic toxins in water [2], [3], [4], [5], [6], [7], [8]. For example, zero valent iron (ZVI) has been successfully used in permeable reactive wall applications to treat chlorinated organic compounds in groundwater [2]. Another promising method noted successful removal of chlorinated ethenes and other related toxins by catalyzation of palladium-on-gold bimetallic nanoparticles [4].

The lifetime of filters can potentially be further enhanced by photocatalysts, which can remove organic contaminants in water more effectively. By irradiating a TiO_2_ electrode with UV light, water splitting was observed via oxidation at the TiO_2_ surface in 1972 by Fujishima and Honda [9]. Microbial cell destruction resulting from TiO_2_ photoelectrochemical reaction was shown in 1985 [10]. Since then, various studies have been conducted to increase the effectiveness of TiO_2_ with respect to photocatalytic decomposition of organic compounds, including photocatalytic sterilization and photocatalytic cancer treatment [8], [11]. Different types of TiO_2_-based photocatalysts including doped, nanostructured and multifunctional composites have been studied [7], [12], [13], and modified materials with a broader optical bandwidth continue to be developed [14]. In the last two decades, efficiency has been enhanced dramatically as a result of the quantization effect by using a nanosized particle reaction [15], [16]. Applying a layer that adsorbs organics to the photocatalyst is a promising step towards obtaining a selectively permeable filter. Furthermore, filtration can then be enhanced by chemical affinity rather than by relying exclusively on size-exclusion, which becomes exceedingly important with smaller diameter contaminants such as viruses. An adsorption layer attracts the toxic pollutants, then free radicals (^•^OH and O_2_^•−^) remove the contaminants with photocatalytic oxidative and reductive reactions, and allow the adsorption layer to regenerate [14], [17], [18]. Nanofiltration membranes made from TiO_2_ are under investigation to eliminate the need for other nanomembranes and the adsorbtive materials [16], [19].

The photocatalyst of interest in this work is an anionic delaminated titanate nanosheet, which is easy to generate and promising for its filtration capability because it is potentially re-usable. Cs-titanate lepidocrocite-analogue parent materials have mainly been used as a source for delaminated titanate nanosheets [17]. Recently, Stewart et al. [13] showed that delaminated sodium nonatitanate (SNT) is as effective as Cs-titanate in the photodegradation of common dyes. It is less expensive to synthesize and is produced in fewer steps. It also performs well in its surface fixed form, which makes it attractive for water treatment technologies. To act effectively in a long lifetime filter, (i) SNT sheets must adhere to surfaces, (ii) viruses must in turn adhere to the SNT sheets, and (iii) viruses must degrade upon exposure to UV illumination. Finally, (ii) and (iii) must be repeated continuously. These functions could be studied using plaque-forming unit assays. However, the inferences made would be indirect. Partial destructive effect by photocatalytic degradation was imaged by transmission electron microscopy [20]. The goal of our work is to establish a technique that can verify these functions directly by imaging without any sample damage.

Atomic force microscopy (AFM) is an attractive technique for meeting this objective. Viruses have been imaged by AFM in both dry and aqueous conditions [21], [22], [23]. Direct imaging by AFM has been used to investigate phage infection of bacteria [24]. Although not yet demonstrated, in principle the SNT nanosheets, the viruses, and their removal can be imaged directly by AFM in the medium of interest. As a first step toward this goal, in this work we have investigated the use of AFM on dry samples. We find that single or multilayer nanosheets are readily adsorbed on mica dipped in a solution to form a cationic glue layer. A hydrophobic coating is used to promote adhesion of phage λ to mica or to the SNT nanosheets. A UV-illuminated SNT sample removes phages λ from the surface when the nanosheets are present, but not in their absence. This work motivates further studies in which virus decomposition is directly imaged in aqueous environment.

## Materials and Methods

### 2.1. Substrate Preparation

**Figure 1:**
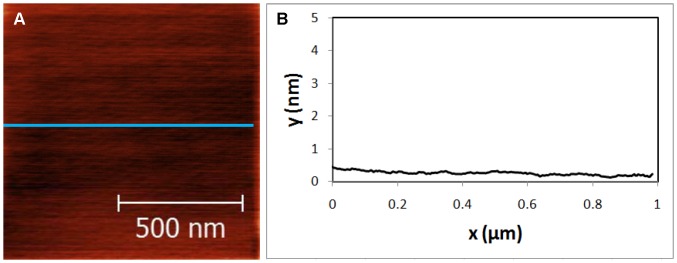
Topography of Cleaved Mica sample. (A) AFM image of cleaved mica surface. (B) Cross sectional image along the blue line shown in (A).

Muscovite mica was selected as a substrate because when cleaved it is smooth on an atomic scale. Thus, virus particles and titanate nanosheets can be clearly observed. Muscovite mica substrates [Ted Pella] were prepared by cleaving 5 cm^2^ samples with the aid of a scalpel blade to expose fresh smooth surfaces. An AFM image of cleaved mica is shown in Fig. 1, indicating a root mean square (rms) roughness of ∼25 pm, likely limited by AFM noise. In the lamellar crystal structure of mica, one of every four Si^4+^ is substituted by Al^3+^ and causes a net negative charge structural surface that is compensated by potassium ions on the interface of mica layers [25]. After cleaving the mica, the surface is expected to have a random charge distribution with neutral average charge.

### 2.2 Adhesion Method

**Figure 2:**
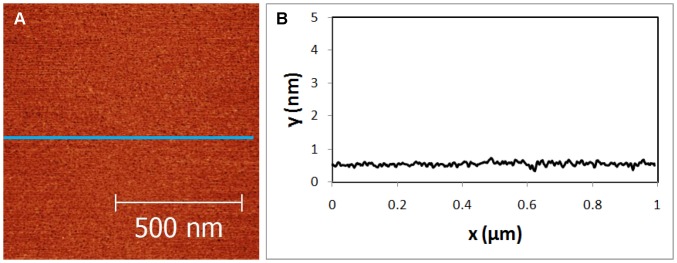
Topography of Mica/GaAl_12_ sample. (A) AFM image of GaAl_12_ adsorbed mica surface. (B) Cross sectional image along the blue line shown in (A).

Titanate nanosheets and viruses are negatively charged in pH 7 water. Surface-bound titanates have a greater potential for practical use in remediating organic and microbial contamination in water and, thus, a cationic adsorption layer is needed. An inorganic cationic ‘glue’ is preferable because photocatalytic reactions would damage organic material. The aluminum tridecamer polycation derivative, [GaO_4_Al_12_(OH)_24_(H_2_O)_12_]^+7^, denoted GaAl_12_, was selected as an adsorption layer [26]. GaAl_12_ carries the highest charge and is most stable in solution of the three available aluminum polycations of this type because it is the weakest acid [27], [28]. To obtain an adsorption layer, a piece of mica was soaked immediately after cleaving in a 1 mg/ml solution of GaAl_12_ prepared according to [27]. After ten minutes, SNT-coated mica was rinsed for approximately 10 s under a gentle flow of ambient temperature water, deionized to a resistivity of 18 MΩ•cm. Mica pieces were then tilted vertically and one edge was contacted by paper toweling to blot away excess liquid. They were then dried in air under ambient conditions with the coated side up. Figure 2 shows a GaAl_12_ coated sample, indicating ∼55 pm rms roughness_._ This smooth coating enables particles to be clearly distinguished particles on the substrate.

### 2.3. Titanate Nanosheets

We used the procedure described prior [13] and it is outlined briefly below. SNT (sodium nonatitanate) was synthesized hydrothermally. Sodium hydroxide (NaOH; 10 g, 0.25 moles) was dissolved in 48 mL DI water in a 125-mL Teflon liner for a Parr reactor. Titanium (IV) isopropoxide (9.6 g, 0.033 moles) was added while stirring vigorously. White slurry formed. The reactor was closed and placed in a 200°C oven for five days. Approximately 4 g of sodium nonatitanate powder was collected by pressure filtration. Proton exchange for Na was carried out with 1 molar HNO_3_ solution, utilizing a Turbula® mixing system (Schatz). Several grams of alkali titanate were combined with 75 mL nitric acid solution in a Teflon bottle for this process. The H+-exchanged powder was collected by centrifugation and washing; first with water then isopropanol. A 40 wt% tetrabutylammonium hydroxide (TBA) aqueous solution was diluted by 50% with water; and the H+-titanate powders were combined with the solution for Turbula® treatment for ∼40 minutes, which resulted in a delaminated colloid. Again, the solid was isolated by centrifugation, washing and drying in a vacuum oven. Finally, the delaminated titanate was re-suspended in water in a stable colloidal form at 1 mg/mL concentration. Mica substrates coated by the GaAl_12_ glue layer were dipped into the prepared solutionrinsed thoroughly with deionized water and dried after coating in a same way as explained in section 2.2.

### 2.4. Virus Solution

MS-2 phage was selected initially because it is nonpathogenic and serves as a model system for immunological, drug delivery and gene delivery studies. Its geometry is spherical with 25 nm diameter, and it has no tail structure. We produced samples successfully. However, phages contracted in air to ∼10 nm in height making them difficult to distinguish from DNA and other particles in the solution that also adsorbed to the mica.

As a result we selected phage λ as a model virus to continue our study [6], [23], [29]. Its diameter is ∼63 nm while its tail is 173 nm long [29]. Its isoelectronic point is pH 4.1, which gives an anionic property to viruses in deionized water. The water used in both the virus preparation and rinsing steps is from the Millipore Direct-Q 3 system (MilliQ). Bacteriophage λ cI857, with a wild type genome length of 48.5 kbp was produced by thermal induction of lysogenic *E. coli* strain AE1. Phage purification details are described elsewhere [30]. All phage samples were purified by CsCl equilibrium centrifugation and dialyzed from CsCl against TM buffer (10 mM MgSO_4_/50 mM Tris⋅HCl, pH 7.4). The final titer was ≈ 10^13^ virions per milliliter, which was determined by plaque assay [31]. The stock solution was diluted 10-fold into the same buffer.

Finally, the solution was ready for deposition. AFM substrate was treated with APTES ((3-Aminopropyl) triethoxysilane) solution and dried with nitrogen gas. During deposition, 40 µl of virus solution was dispensed on the APTES-coated substrate for 30 minutes. In the last step the mica was rinsed with 4–5 ml of deionized water at ambient temperature and a resistivity of 19 MΩ•cm. Then the virus-coated mica was dried in air under ambient conditions with the virus-coated side up.

### 2.5. UV Exposure

Some samples were exposed to UV light (15 Watt and 254 nm wavelength) for 15 minutes at a 15 cm distance from a light source. UV illumination of the SNT nanosheets generates ^•^OH and O_2_^•−^ free radicals, which degrade organic material. This UV source was chosen because it is a small fixture of a type commonly present in laboratories where aseptic processing takes place, such as for microbiology, cell culture, virology, and genetic engineering. The distance from source to sample was chosen to maximize the number of specimens that could be exposed at the same time in a field of sufficiently uniform irradiance. This had been determined in earlier work with photocatalysis of organic dye compounds [13]. As such, these conditions are not proposed for industrial scale-up, which could achieve great improvement, but simply a laboratory demonstration of the principles of interest to the study.

### 2.6. AFM Operation Procedure

Tapping mode AFM in air was used to observe viruses and SNT. In this technique, a cantilever operates near its resonant frequency and lightly taps on a soft sample with reduced lateral forces compared to contact mode AFM [32]. Veeco 3100 and Agilent 5500 microscopes were used. Cantilevers (Nanosensors, Model PPP-NCR) with a nominal tip radius of 10 nm, a resonant frequency of 330 kHz and a spring constant was 40 N/m were used. A softer cantilever (Olympus, Model AC240TS) with nominal tip radius of 9 nm, 80 kHz resonance frequency and 2 N/m spring constant was also used. The excitation frequency was reduced until the tip oscillation amplitude was 90% of its maximum amplitude. In order to tap gently, the set point was increased until trace and retrace were close to losing engagement, but without sacrificing image quality. The heights of viruses on the same samples did not depend on the cantilever type, suggesting that indentation of the tip into the viruses was insignificant. The tip radius was measured by scanning standard 30 nm gold particles (PELCO Atomic Force Microscopy Gold Calibration Kit) according to [33]. Typical radii were in good agreement with the manufacturer’s specifications and no change was observed before and after imaging. Images were prepared with Gwyddion software by making a plane-fit adjustment to the raw data.

## Results and Discussion

**Figure 3:**
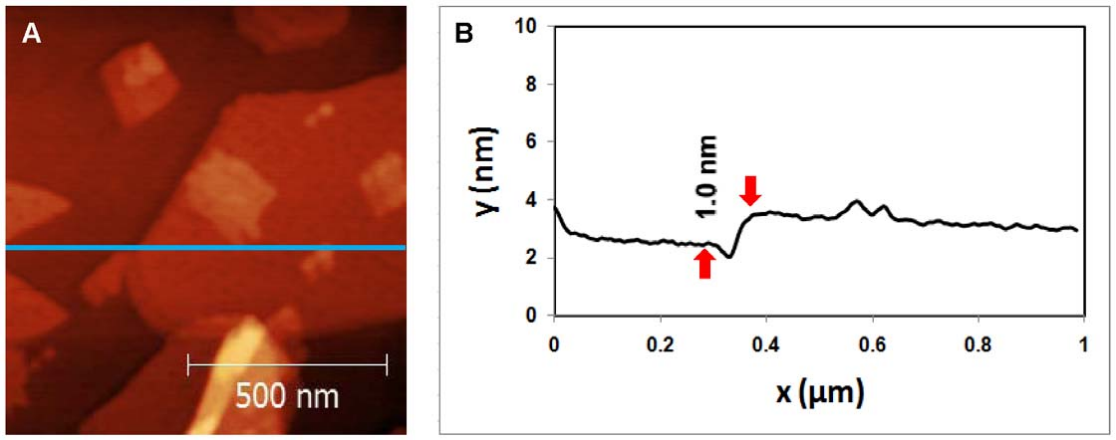
Topography of Mica/GaAl_12_/Nanosheets sample. (A) AFM image of delaminated SNT adsorbed mica surface. (B) Cross sectional image along the blue line shown in (A).

**Figure 4:**
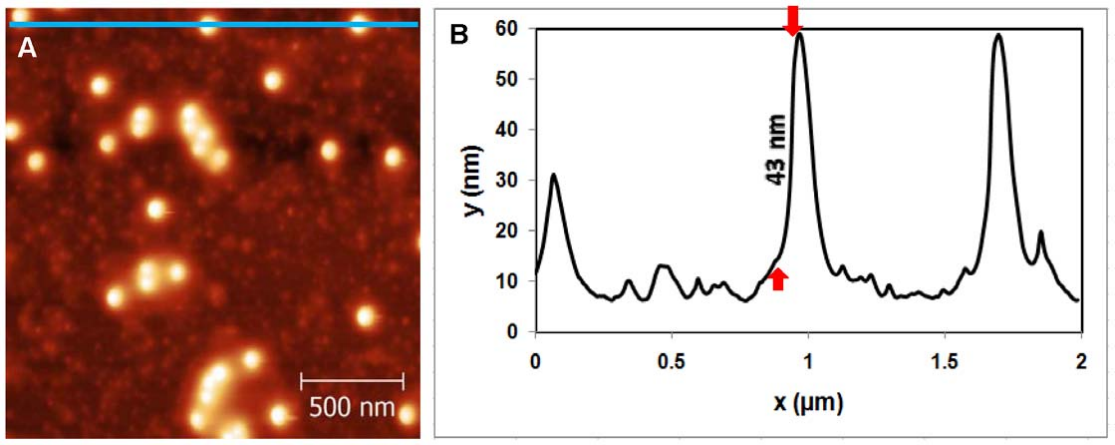
Topography of Mica/GaAl_12_/APTES/Phage λ/UV sample. (A) AFM image of virus solution adhered on mica after the 15 min UV exposure. (B) Cross sectional image along the blue line shown in (A).

**Figure 5:**
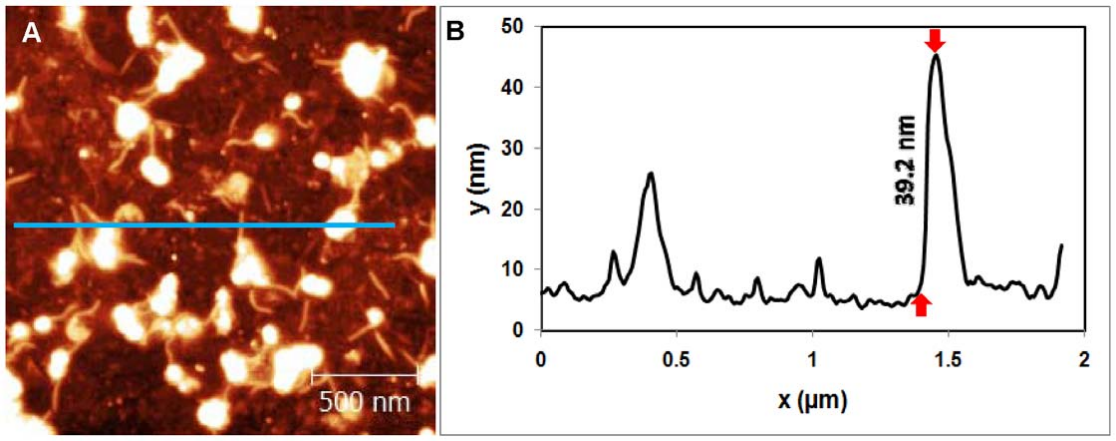
Topography of Mica/GaAl_12_/Nanosheets/APTES/Phage λ sample. (A) AFM image of virus solution adhered on SNT coated mica. (B) Cross sectional image along the blue line shown in (A).

**Figure 6:**
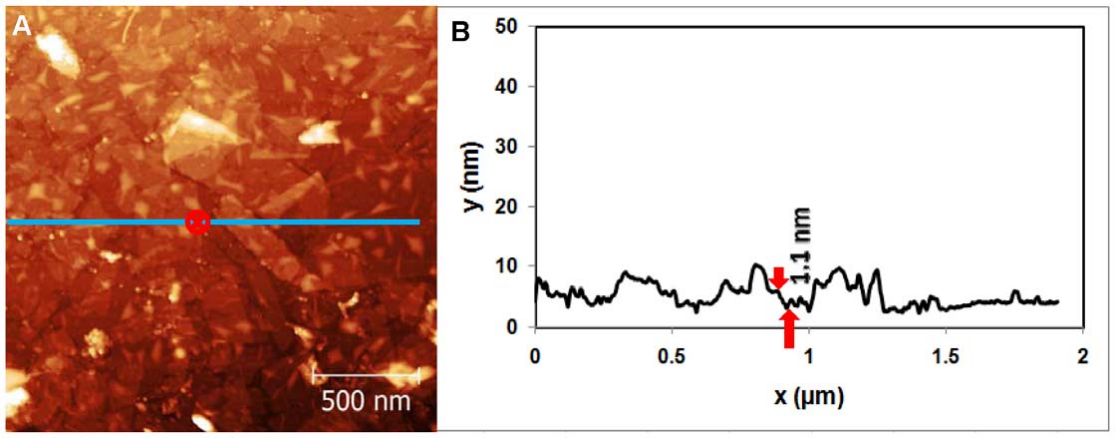
Topography of Mica/GaAl_12_/Nanosheets/APTES/Phage λ/UV sample. (A) AFM image of virus solution adhered on SNT coated mica after the 15 min UV exposure. (B) Cross sectional image along the blue line shown in (A).

**Figure 7:**
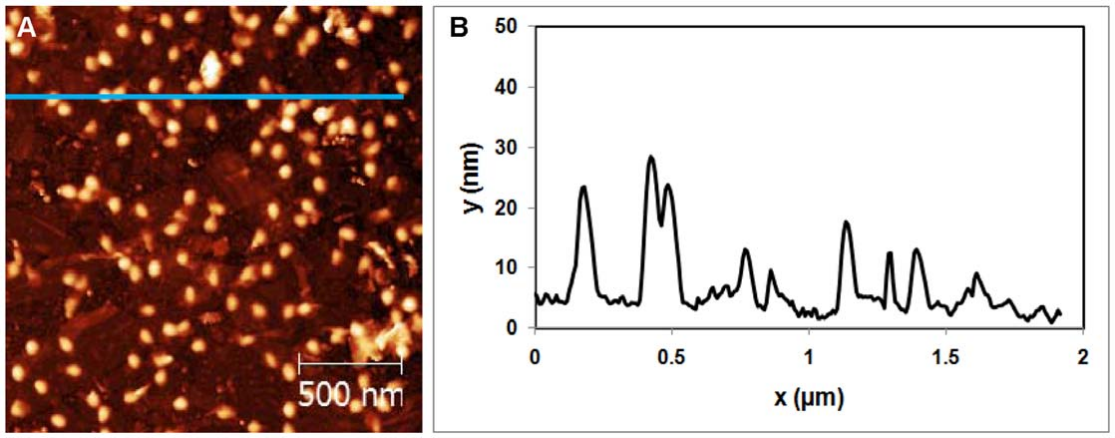
Topography of Mica/GaAl_12_/Nanosheets/GaAl_12_/APTES/Phage λ/UV sample. (A) AFM image of virus solution adhered on SNT coated mica with GaAl_12_ layer on top after the 15 min UV exposure. (B) Cross sectional image along the blue line shown in (A).

Table 1 describes the treatments for samples imaged in Figures 2, 3, 4, 5, 6, 7. Each figure is representative of images taken in five or more areas of a given sample. These samples are discussed in the following paragraphs.

**Table 1:** The treatments for samples imaged in Figures 2, 3, 4, 5, 6, 7.

Figure	Description
2	Mica/GaAl_12_
3	Mica/GaAl_12_/Nanosheets
4	Mica/GaAl_12_/APTES/Phage λ/UV
5	Mica/GaAl_12_/Nanosheets/APTES/Phage λ
6	Mica/GaAl_12_/Nanosheets/APTES/Phage λ/UV
7	Mica/GaAl_12_/Nanosheets/GaAl_12_/APTES/Phage λ/UV

First, mica samples were covered by the cationic glue layer, dipped in the colloidal SNT solution, dried and imaged. SNT single and double layers are clearly observed in Fig. 3(A). The thickness of an individual layer is 1 nanometer, as seen from the line profile in Fig. 3(B). This compares well with the expected thickness of ∼1.0 nm [13]. Roughly 50% of the surface is covered by the nanosheets in this image, but typically the coverage was 90% or greater (this particular area was chosen to highlight the nanosheets clearly). In general, most areas are covered by double layers, and occasionally by triple or quadruple layers, which become more difficult to resolve (see Fig. 11(b) of [Stewart et al.]). A more typical area showing the general morphology of the SNT nanosheets is seen in Fig. 6(A) below.

Next, phage λ was adsorbed onto the mica (with or without the glue layer) and the APTES coating. The virus samples had a distinguishable structure with spherical capsid and tail; however, tails were not observed in all trials. We measured a viral capsid height of 30–45 nm in air. In an air environment, viruses typically contract by 30% from their size in an aqueous medium [21], [22]. The measured capsid heights are in reasonable agreement with this value.

Variables were now examined to probe the contribution to the degradation mechanism. An example of phage λ adsorbed onto mica with a glue layer and an APTES coating, and then exposed to UV is seen in Fig. 4(A). Figure 4(A) shows intact adsorbed λ phages. As shown in Fig. 4(B), the phage height is 43 nm, which is consistent with the virus sample imaged in air. Similar images were obtained on samples that were not exposed to UV illumination. This demonstrates that UV-exposed phages λ do not degrade perceptibly when adsorbed to the mica substrate.

In order to test the effectiveness of the SNT in degrading viruses, it is first necessary to adsorb them onto SNT. Mica was coated with titanate nanosheets, then the APTES coating was applied. A drop of the virus solution was spread over the sample, and then rinsed and dried. Viruses and their tails are readily observed in Fig. 5(A). The virus capsid height is ∼40 nm as seen in Fig. 5(B), in good agreement with Fig. 4(B). In Fig. 5(A), the titanate nanosheets are present but cannot be clearly distinguished because they are covered by the phages λ.

Next we exposed a sample treated in the same way as in Fig. 5 to UV light for 15 minutes before drying. The SNT nanosheets are now clearly distinguishable, as seen in Fig. 6(A). Some small particles are also seen in this image, but these are also often observed in samples where only SNT was adsorbed. Figure 6(B) indicates a 1.1 nm nanosheet layer thickness among multilayer scattered titanate nanosheets. This directly demonstrates that the sodium nonatitanate sheets are effective in degrading phage λ in water.

Finally, we tested SNT nanosheet performance with an additional Ga Al_12_ glue layer on top of the titanate layer before the APTES was adsorbed. The idea was to attract as many virus particles as possible to the surface with the combination of the cationic glue layer and the hydrophobic layer. These samples showed slightly damaged viruses after UV treatment as seen in Fig. 7. This result suggests that the extra inorganic glue layer prevents free radicals from reaching the virus particles, and implies that the inorganic layer remains when exposed to UV light. As a result, the GaAl_12_ layer, which is used to glue the SNT sheets to the mica, survives the UV illumination, and therefore the nanosheets can be expected to remain in place when reused.

### Conclusions

SNT delaminated sheets deposition technique was confirmed by AFM. Single and multiple layers were observed on the substrate with 1.0–1.1 nm layer thickness. Phages λ were scattered on the substrate distinguishably. Virus degradation was shown by AFM when SNT was illuminated by UV radiation, yet they retained their integrity upon UV irradiation when the titanate nanosheets were not present. This study used virus particles as a model microbial contaminant, and the results suggest that the same or similar materials might be employed effectively in degrading bacteria and biofilms that are problematic in membrane filter systems. Future work includes studies demonstrating utility and optimization of SNT, including comparison with other photocatalysts. Reusability, which is very crucial at water filtration, will be investigated. Further studies in aqueous medium are of interest. They will enable quantification of virus degradation rates, and perhaps will give insight into degradation mechanisms.
